# A passive blood separation sensing platform for point-of-care devices

**DOI:** 10.1038/s44328-025-00038-x

**Published:** 2025-05-02

**Authors:** Cameron Gilroy, Callum D. Silver, Casper Kunstmann-Olsen, Lisa M. Miller, Steven D. Johnson, Thomas F. Krauss

**Affiliations:** 1 Hull York Medical School, Siwards Way, University of York, Heslington, York UK; 2 School of Physics and Technology, University of York, Heslington, York UK; 3 Mads Clausen Institute, University of Southern Denmark, Sonderborg, Denmark

**Keywords:** Diagnostic markers, Applied optics, Nanobiotechnology, Lab-on-a-chip, Nanobiotechnology, Sensors and probes, Biomedical engineering

## Abstract

The blood test is one of the most performed investigations in clinical practice, with samples typically analysed in a centralised laboratory. Many of these tests monitor routine conditions that would benefit from a point-of-care approach, reducing the burden on practitioners, patients and healthcare systems. Such a decentralised model requires the development of sophisticated, yet easy-to-use technology; however, platforms that combine high-performance with low-cost and simplicity remain scarce. Moreover, most research papers only address a subset of requirements and study specific aspects in isolation. A systems approach that considers the interplay between each element of the technology is clearly required to develop a coherent solution. Here, we present such a systems approach in the context of testing for C-reactive protein (CRP), a commonly requested test in clinical practise that indicates inflammation and is particularly relevant for monitoring patients with chronic diseases, e.g. those with rheumatoid arthritis or who are undergoing cancer therapy. The approach we take here features an entirely passive microfluidic cartridge for blood separation, integrated with a high-performance sensing platform which we have tested in a real-world context. The device is compatible with a handheld detection unit and is simple to use yet can accurately detect CRP levels at clinically relevant levels.

## Introduction

A recent report on the future of point-of-care (POC) and rapid testing^1^ predicts that diagnostic technologies will improve in accuracy and speed and that they will become easier to use and interpret. This development is essential for mitigating the workload of healthcare professionals so they can cope with the ever-increasing healthcare burden posed by an ageing society. In addition, POC devices have the potential to improve care by increasing convenience and speed of diagnosis and, when used by patients, increase motivation to better manage their condition^2,3^.

To support this trend, the research community needs to focus on technologies that are easy to use, can be built with low-cost components yet do not compromise on clinically required performance. Moreover, researchers should consider a systems approach whereby a given technology is designed and assessed in a real-world context rather than focussing only on individual parameters in a laboratory setting.

Here, we present an example of such a systems approach in the context of testing for C-reactive protein (CRP) from clinical blood samples. Our work is based on the guided mode resonance (GMR) photonic sensor that we have developed previously and where we have already demonstrated very low limits of detection for protein biomarkers, i.e. pg/mL levels of troponin, procalcitonin and C-Reactive Protein in urine^4^ and IL-6 and TNF-α in wound fluid^5^, as well as operation of a handheld instrument^6^. We now show the integration of this technology with an entirely passive microfluidic cartridge and demonstrate the response to CRP levels in the blood of real patients.

CRP is a pentameric protein that is synthesised by the liver in response to inflammation, tissue damage, infection and malignant neoplasia^7^. In healthy individuals, the median concentration of circulating CRP is 0.8 mg/L. Please note that we use mg/L here as the unit of measurement to reflect clinical practice, which is equivalent to the µg/mL unit more commonly used in biosensor research.

The level of CRP in blood can rise by a factor of hundred to a thousand^8^ following pathological insult. CRP levels are also used to guide treatment for patients with autoimmune disease or to indicate antibiotic therapy^9^. It follows, therefore that obtaining test results on a simple instrument and in a timely manner can reduce time to treatment, prevent the progression of a condition, and allow for monitoring away from a clinic. Moreover, delays between blood sampling and molecular testing is a well-recognised cause of red cell rupture (haemolysis) which can render results unreliable^10^, so reducing time to analysis is highly desirable.

A CRP test needs to be conducted in separated plasma to eliminate contamination from cells and the products of their degradation. In laboratories, separation is accomplished by centrifugation, which conventionally is not compatible with a bedside test and typically occurs several hours after the sample has been obtained from the patient.

A near-patient test, therefore, needs to include a mechanism for separating blood into its constituent components. Many solutions have been developed to this end, involving various materials and separation methods. Several studies propose paper-based platforms, employing strategies such as inducing blood agglutination using antibodies against erythrocyte surface antigens^11,12^ or membrane separation^13–15^ with advantages including cost-effectiveness, universal availability and compatibility with a wide range of printing techniques^13^. Others rely on microfluidic designs which exploit hydrodynamic forces^16,17^ or actuators driven by magnetism^18,19^ or acoustics^20^. Most of these require an external force to drive the movement of fluid such as a pump.

Commercial POC sensors are, of course, available but few are sufficiently simple that they can be operated by a non-specialist, relying instead upon complex procedures or expensive analysers^21^. For a POC testing device to reach widespread use, simplicity and high performance are a necessity and cost-effectiveness is essential^22^. We are not aware of another solution that uses an entirely passive microfluidic approach comparable yet is integrated with a high-performance sensing platform that can reach very low limit of detection^4,5,23,24^ as well as showing responsiveness to clinically relevant levels of a biomarker in blood, as we show here.

## Results

### Cartridge design

**Fig. 1:**
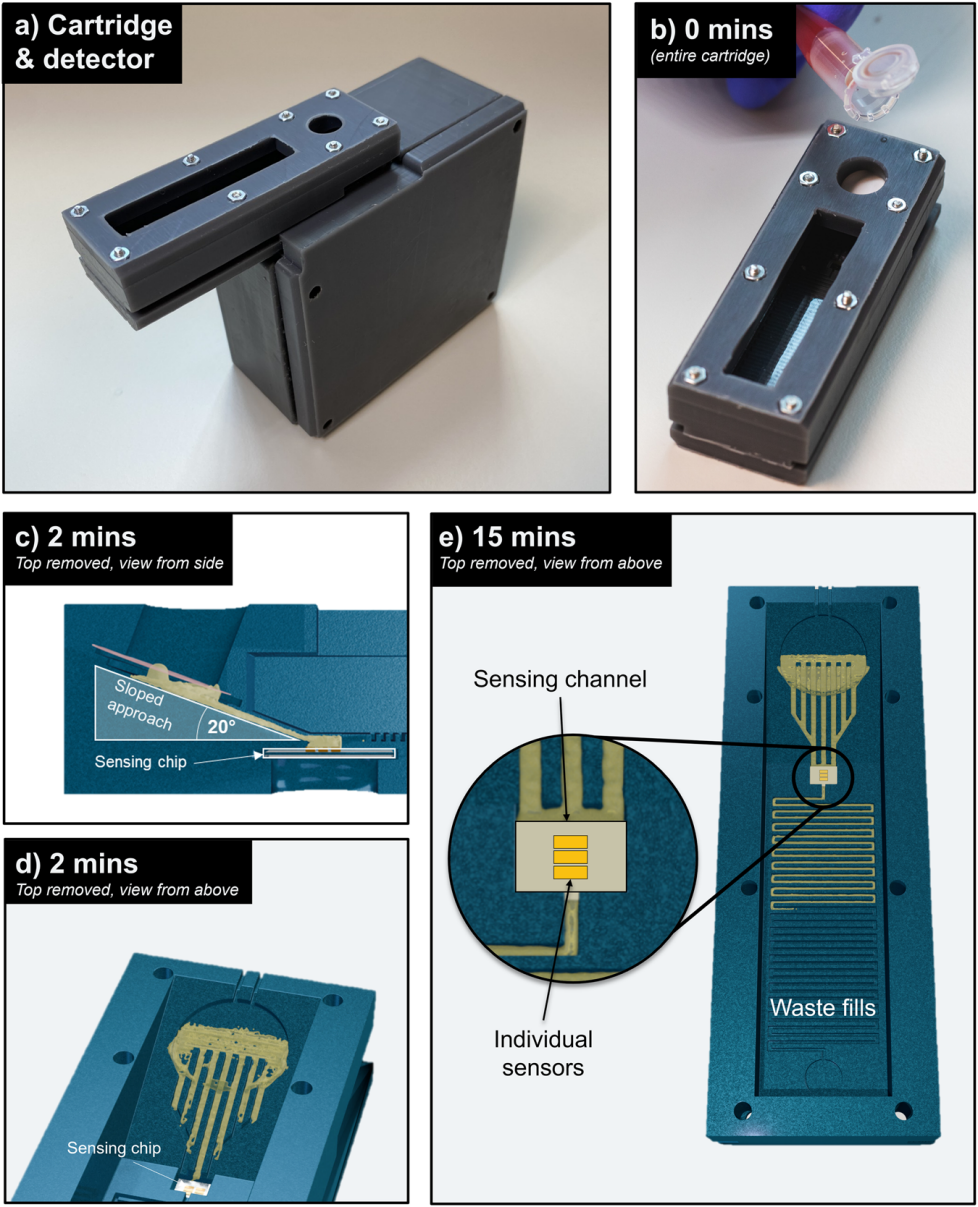
Form and operation of the blood filtering device. **a** 3D printed sensing platform and detection unit. **b** Blood sample is added to the inlet. **c**, **d** A sloped approach enables the gravity driven flow of blood plasma. **e** Plasma flows over the sensor and accumulates in a waste microfluidic channel.

The cartridge is shown in Fig. 1a, b and consists of three separate components, each printed by 3D stereolithography. The top component acts as an inlet to which 0.5 mL of blood solution can be added where it then meets a plasma separation disc, (Vivid^TM^, USA). The motivation behind and use of membranes for microsampling has been recently reviewed elsewhere^25^.

The blood is pushed through the membrane by gravity Fig. 1c, d and the resulting plasma is wicked from the back membrane surface by 800 × 750 µm channels, which direct the resulting plasma towards the sensing chip. The liquid wets the sensor and is then drawn across it by a microfluidic pump that consists of a meandering channel, i.e. the pumping action is due to capillary forces, Fig. 1e. The cross-section of the channel determines the flow rate and together with the length of the channel, it determines the total volume of liquid that can be drawn across the sensor. We designed the dimensions of the capillary channel, 500 × 750 µm cross-section, to match its flow rate to the 0.5 mL sample volume and time required for the antibody-antigen binding to saturate, which is of an order of 12–15 min. We calculate the effectively achieved flow rate to be ~24 µL min^−1^ which is constant across the measurement period. We include a video of the operation of the device, see Supplementary Movie 1.

We note that many other microfluidic approaches have previously been introduced to drive the flow of analyte, yet they tend to use a horizontal geometry^26–28^. It is very difficult, however, to match the fluidic impedance of all sections, given, e.g. the variation in surface area between the filter, the intermediate channel and the sensor. We address this issue by introducing an angled section between the filter and the sensor, which ensures that the fluid is drawn towards the sensor and into the capillary pump without external actuation. We use an angle of 20° to create a gravity-driven flow of analyte. This design enables the completely passive separation and delivery of blood plasma to the sensing area. We include a schematic of all components of the device in Supplementary Fig. 1.

The cartridge is compatible with a 3D printed detection unit, Fig. 1a containing all required optical components (Supplementary Fig. 2) within a compact box which we have previously described^6^. It is important to highlight that all measurements were conducted on a standard laboratory bench and with a handheld instrument, thereby mimicking the environment encountered in clinical practice.

### Separation of blood solution

Finger-prick blood samples were obtained from volunteers and diluted in phosphate buffered saline (PBS) with 1 mM ethylenediaminetetraacetic acid (EDTA) to inhibit clot formation. We collect 10 µL of blood via pipette from a lanced finger, a volume we could reliably extract from a finger-prick sample, made up to 600 µL in PBS to yield a 1 in 60 solution. We note that this blood volume is within the limit of the filtering capacity of the membrane which, for the 17 mm diameter disc used here, would be approximately ~40 µL of whole blood.

**Fig. 2:**
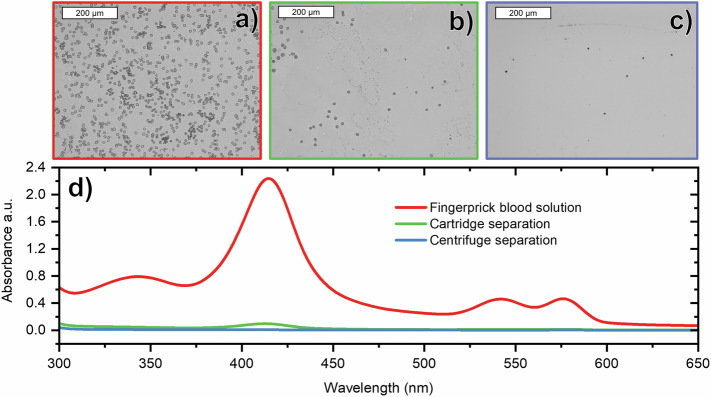
Blood filtering capability of the device is measured by optical microscopy and absorption spectra measurements. Optical micrographs of **a** Blood in buffer solution. Particle counting calculates 370 cells per 1000 µm^2^. **b** The same sample after passing through the cartridge showing 12 cells per 1000 µm^2^, a 97% reduction. **c** Plasma collected from a centrifuged sample showing 3 cells per 1000 µm. **d** Absorption spectra of blood and plasma solutions derived from different separation methods show that the cartridge approach performs almost as well as a centrifuge.

To characterise the separation performance of the cartridge, we compare micrographs of blood spots from an untreated solution to spots from a solution that has passed through the cartridge, Fig. 2a, b. 500 µL of solution was added to the inlet, then collected via syringe from the sensor well, thus it is representative of the fluid in contact with the sensor. Whilst the reduction in the quantity of red blood cells is clear from the micrographs, an estimate of the cell density was made using a particle counting programme in image processing software, ImageJ®. The untreated solution showed a density of approximately 370 cells per 1000 µm^2^ compared to the filtered solution at 12 per 1000 µm^2^, a 97% reduction.

In addition, we compare the absorption spectra of blood and plasma derived by different separation methods, Fig. 2c. The peak at 415 nm is a Soret band and that at 542 and 576 nm corresponds to β and α bands of the oxygenated haemoglobin porphyrin ring respectively^29,30^. As haemoglobin is found within erythrocytes, the intensity of these bands can act as a proxy for the presence of red blood cells *or* haemolysis within the filtered plasma.

As expected, the solution of unfiltered blood solution has the greatest absorption at these characteristic wavelengths. The plasma obtained from our cartridge device shows far less absorption and is close to that of a plasma sample prepared by the gold standard method, centrifugation. There remains a small peak at 425 nm which is consistent with the low concentration of red cells observed in the micrograph of Fig. 2b.

The ratio of absorbance intensity at 415 nm was found to be 370:15:1 for *blood:cartridge:centrifuged* sample. This would indicate a 96% reduction in haemoglobin concentration between the cartridge separated blood solution and the same concentration of the untreated blood solution.

To estimate the extent of protein loss within the fluidic manifold, we compare the concentration of CRP in a blood sample separated by the cartridge to that separated by centrifugation. We prepare samples from finger-prick samples obtained from a healthy volunteer: one unadulterated, which we assume to have a CRP concentration of <1 mg/L and the others spiked to concentrations of 10 mg/L, 50 mg/L and 200 mg/L. The blood solution was diluted to 1 in 60 in PBS, passed through the cartridge system and the resulting plasma was extracted from the recess immediately above the sensor via a pipette. The centrifuged sample was similarly diluted and spun at 1500 rpm for 10 min and the top plasma layer extracted by pipette.

**Fig. 3:**
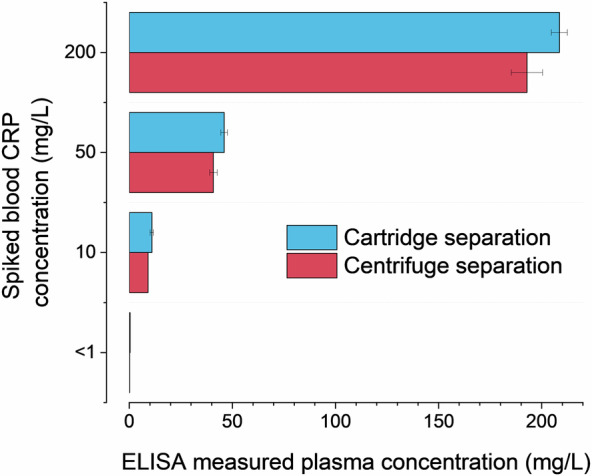
CRP concentration was measured in plasma solution derived from whole blood separated by the cartridge device and a centrifugation via enzyme linked immune immunosorbent assay (ELISA). There is no significant loss of analyte

We then measure the CRP concentrations of these plasma solutions using an enzyme linked immunosorbent assay (ELISA), Fig. 3. It shows that the samples processed via the cartridge and centrifuge maintain comparable CRP concentrations.

### Sensing platform

The sensing chip is directly integrated into the cartridge as shown in Fig. 1e. We use the ‘chirped’ GMR modality with a grating fabricated in Si_3_N_4_ on borosilicate glass as previously described^31^. Illumination of the grating with polarised, collimated light excites a guided mode resonance in which light is coupled into a guided mode in the high refractive index silicon nitride layer and is coherently back-reflected. The interference between the transmitted and the reflected light then determines the phase and amplitude of the reflected signal.

The spectral resonance of a uniform GMR grating is usually determined with a spectrometer. Using the chirped geometry, in which the period of the grating varies spatially, we translate spectral into spatial information, allowing the resonance to be read out by imaging alone. This approach eliminates the need for a bulky and expensive spectrometer, requiring only a low-cost CMOS camera for the readout.

**Fig. 4:**
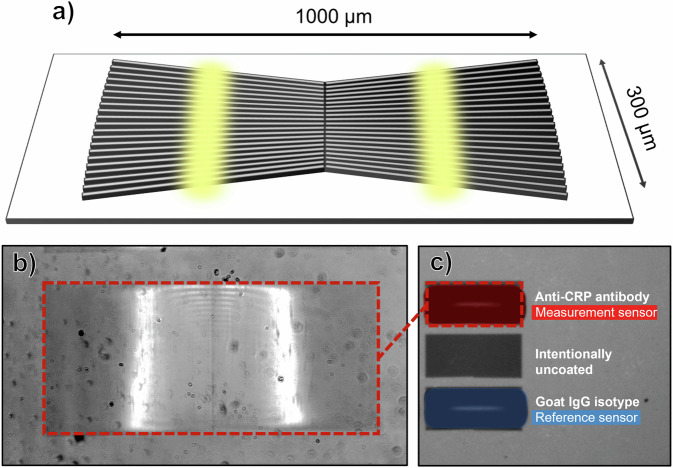
The sensing strategy of the device is functionalisation of a guided mode resonance sensor. **a** Schematic of the silicon nitride ‘chirped’ guided mode resonance sensing chip. The sensor consists of two gratings in a ‘bowtie’ configuration to provide immunity to mechanical noise. The grating spacing here is exaggerated for illustrative purposes. **b** Experimental image captured of the sensor under monochromatic illumination. **c** Functionalisation of the top and bottom sensors with anti-CRP and goat IgG isotype antibodies, respectively, which we designate as measurement and reference sensors, respectively.

Each sensor consists of a pair of chirped GMR sensors in a ‘bowtie’ configuration, as shown in Fig. 4a, b^24,32,33^. This arrangement enables mechanical vibrations to be removed from the measured sensor signal, as is essential for a handheld device, since the resonance position (bright bars in Fig. 4a, b will both move in the same direction if there is mechanical motion, but in opposite directions when there is a local change in refractive index^23^. Thus, by measuring the separation of the resonance positions, shifts not associated with refractive index changes can be eliminated, thereby making the readout tolerant against mechanical vibrations.

Three sets of bowtie sensors are arranged on the substrate as shown as in Fig. 4c. The entire substrate is first functionalised with a polydopamine layer to enable covalent attachment of antibodies to the sensor surface. Next, using a precision dispensing system *(sciFLEXARRAYER S3, Scienion®)*, the top sensor is spotted with capture antibodies against human CRP *(Scripps Laboratories, GC019) and acts as* a measurement sensor for the binding of the target analyte. The bottom sensor was functionalised via spotting with goat IgG isotype control *(Invitrogen 02-6202)* which lacks specificity to the target analyte and therefore acts as the reference sensor. Using confocal fluorescence microscopy, we confirm the binding of CRP with its antibody on the sensor surface, see Supplementary Fig. 3.

**Fig. 5:**
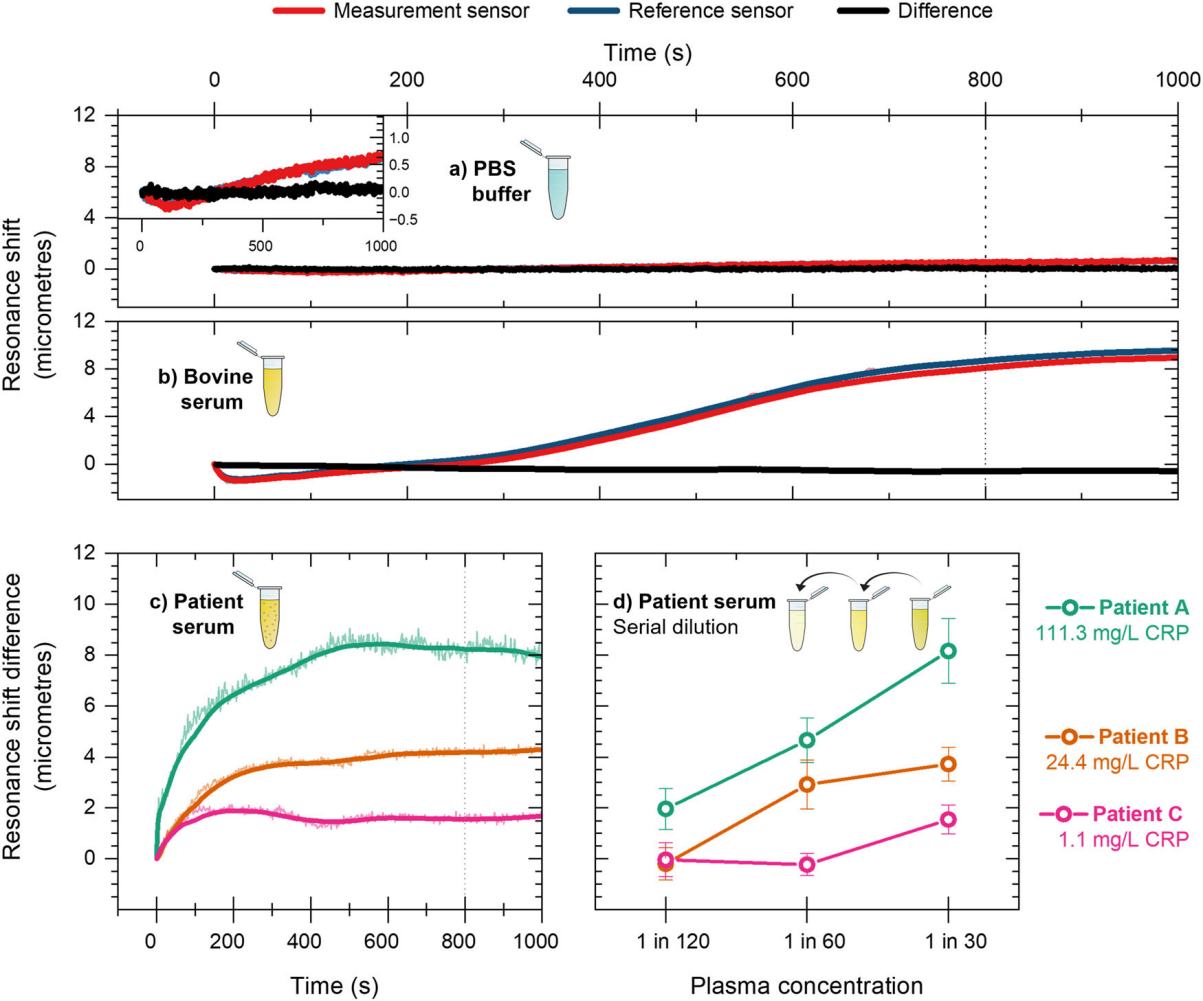
Sensing CRP in samples obtained from patient plasma samples. **a** Resonance shift over time for the measurement and reference sensors in a PBS buffer solution. The error in the difference between sensors is approximately 0.10 microns. The inset shows same data on a smaller vertical scale. **b** In bovine serum, the measurement and reference sensors exhibit a binding curve associated with non-specific protein adsorption to the sensor surface however, the difference between sensors is also negligible, with an error of 0.70 microns. **c** The difference in resonance shift between the measurement and reference sensors for three patient serum samples in a 1 in 30 solution. **d** Serial dilution of patient serum samples showing increasing difference in resonance shift correlates with decreased dilution.

To determine the measurement noise level of the cartridge system, we initially measured the response of the sensor when challenged with PBS buffer, Fig. 5a. As expected, the reference and measurement sensors show a near-identical response to the PBS solution, both reacting equally to the change in bulk refractive index and other environmental changes such as temperature. Since we measure the sensor response as the distance between resonance positions on the bowtie geometry, we typically record the sensor response in micrometres. We note a small drift in both the measurement and reference sensors, which is most likely due to thermal drift, but the difference results in a flat line with a noise of 0.11 microns root mean square (RMS) (equivalent to 0.8 picometres wavelength shift).

Next, we assess the effect of non-specific binding ('fouling') to the sensor surface by testing the system with a solution of foetal bovine serum, a substance which is devoid of human CRP. Fig. 5b shows the corresponding shifts for the measurement and reference sensors and their difference. We clearly see some non-specific binding, but again, the difference in response between the measurement and reference sensors is minimal, although the noise value has gone up to 0.70 microns RMS. As an additional control, we also functionalise both sensors with the isotype antibody which yields a plot whose difference is also approximately zero, see Supplementary Fig. 4.

Having verified the basic operation, we are able to move on to real, clinical samples. Plasma samples were donated by inpatients at the York and Scarborough Teaching Hospitals NHS Foundation Trust, York, UK. The Trust also determined CRP levels using a particle-enhanced immunoturbidimetric assay. Note that these samples were already centrifuged at the hospital, so the blood filter was not strictly needed, yet we used the same cartridge as described above for consistency. Measurements using blood solution will be discussed in the following section. From the range of samples we received, we selected representative examples with CRP concentrations of 113.3 mg/L (*Patient A*), 24.4 mg/L (*Patient B*) and 1.4 mg/L (*Patient C*) to cover the clinically relevant range.

Fig. 5c shows the sensor response to these three patient samples following dilution in PBS buffer to a ratio of 1 in 30. In contrast to measurements with PBS and bovine serum (Fig. 5a), the differential shift is now non-zero, exhibiting a clear binding curve in all cases. The magnitude of the differential shift correlates with the concentration of CRP in the sample.

Using the same method, we also measured the sensor response for three clinical samples at a range of dilution ratios. As can been seen in Fig. 5d, the magnitude of the observed resonance shift decreases with increasing dilution, which is expected as the CRP concentration is reduced.

### Fingerprick blood

Lastly, we demonstrate the full capability of our device by measuring CRP in blood solution. Samples were obtained from healthy volunteers via finger-prick and diluted to 1 in 60 in a 1 mM solution of EDTA in PBS buffer. We initially determine the CRP concentration in the unadulterated blood sample of the volunteer via ELISA. The CRP concentration was found to be at normal physiological levels, i.e. <1 mg/L in an undiluted sample.

**Fig. 6:**
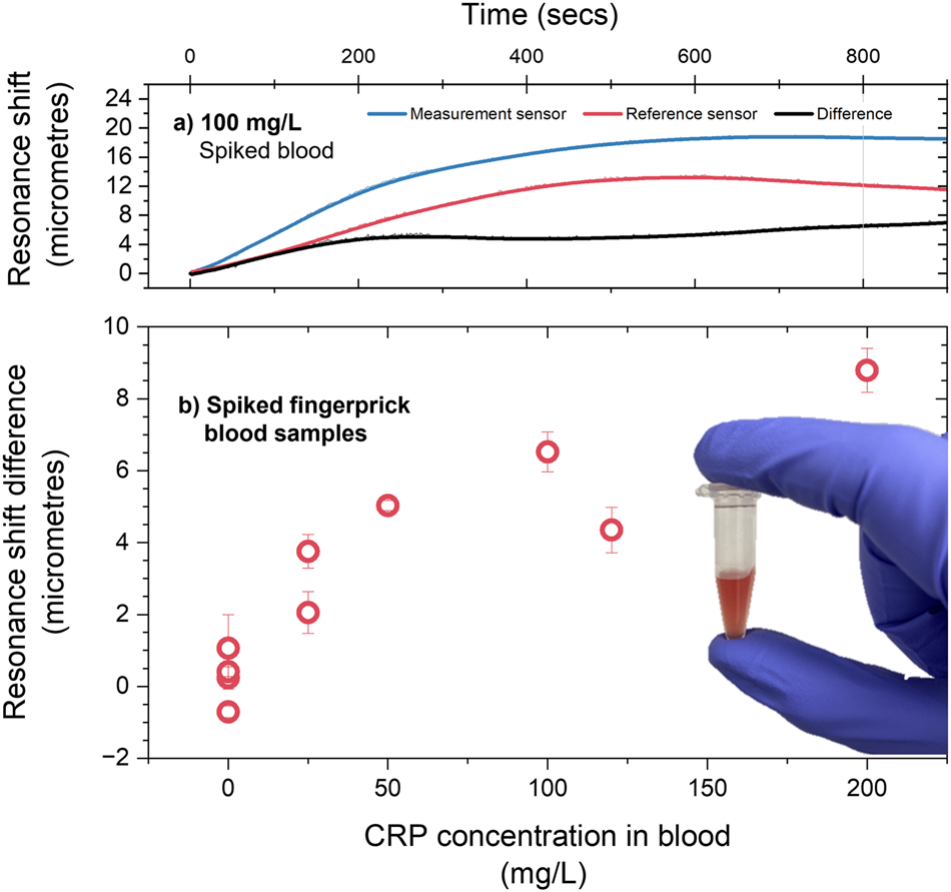
Sensing CRP in blood. **a** Shift in the measurement and reference sensor resonance position and their difference for a 100 mg/L spiked blood sample. **b** Resonance shift difference for spiked fingerprick blood samples across a clinically relevant range.

We then measured the response of our cartridge system to both the unadulterated sample and those spiked to pathological levels of CRP, i.e. a range of concentrations up to 200 mg/L. As before, 0.5 mL of the blood solution was added to the cartridge inlet, and the sensor was consequently exposed to filtered plasma. Fig. 6 shows the response of the sensor to these solutions. We show the 100 mg/L sample response as an example in Fig. 6a.

Fig. 6b shows the resonance shift difference for increasing CRP concentration in the finger-prick bloods samples. There is a trend of increasing resonance shift difference with increasing concentration, as would be expected. The change in shift difference is greatest at lower concentrations and slows as concentration increases, presumably consistent with saturation of binding sites on the measurement sensor.

## Discussion

We present a sensing platform which integrates a high-performance and label-free optical sensor with a fluidic cartridge that provides entirely passive blood filtration and capillary force pumping for sample preparation. The key advantage of the platform lies in its low cost and simplicity, both in terms of design and operation, without compromising on diagnostic performance.

The passive nature of our approach obviates the requirement for more expensive and complex arrangements to drive fluid movement, as does the lack of labelling or washing steps. This simplicity is enabled by using gravity to initiate the fluidic flow and to drive it through the filter, followed by a capillary pump to control the flow across the sensor chip. The combination of gravitational and capillary forces provides an elegant solution to the mismatched fluidic impedance of the various sections.

The simplicity extends to the use of a single fluidic channel with multiple, differently functionalised sensors, which allows for signal and reference to be measured concurrently using the same light source and in the same field of view of the camera. This ensures that both sensors experience the same measurement and environmental noise, which can then be subtracted. Looking forward, this arrangement can also support the detection of more than one disease biomarker by functionalisation of multiple sensors with antibodies directed against different biomolecules, a technique we have demonstrated previously in urine^4^, albeit using multiple fluidic channels. This approach could also form the basis of internal quality control based on the concordance of multiple control sensors.

Lastly, we consider how an end-user may interact with this sensing platform, especially in the context of a POC device. Whilst its use does require a degree of sample processing in the form of dilution, we have seen with COVID-19 lateral flow assays that simple preprocessing steps are acceptable to untrained users. In our Supplementary Movie, we show how the sample can be obtained with inexpensive, fixed-volume, disposable double-bulb pipettes. We also acknowledge the development of commercially available microsampling companies who show promise in patient-collected blood samples^34,35^.

Additionally, the symmetrical resonance structures of the bowtie sensor arrangement render the device largely immune to positional changes and mechanical vibration and other environmental stimuli which many other optical techniques must account for with robust mechanical designs that preserve optical alignment. Because of this, the sensing platform is intrinsically stable; indeed, we have conducted all our experiments on a lab bench as opposed to a stabilised optical table.

Future work on our device should carefully consider how differences in blood composition between patients can be adequately accounted and corrected for. For example, the proportion of blood which is constituted by red cells (haematocrit) can vary widely in disease states. If haematocrit is reduced, as in anaemia, the plasma concentration will underestimate the total blood concentration and if is increased, as in polycythaemia, it will overestimate. Here we have analysed both venous and capillary blood samples, however, we note that available evidence suggests CRP concentrations are comparable between the two sampling methods^36,37^.

As a result, estimations of biomarker concentrations which are derived from separated plasma can over- or under-estimate the true concentration within blood unless it is corrected for. Indeed, it has been recognised that haematocrit variation causes large distortions in glucose readings in commercial devices which do not employ correction methods^38^. POC devices which can measure these haematological parameters have been described elsewhere^39,40^ and accurate measurement of any blood analyte will require integration with these strategies.

Improving the quantification capabilities of our sensing platform will require not only this consideration, but also the optimisation of other key parameters. For instance, it follows that a reduction in the binding of non-target molecules, fouling, will increase our analyte signal. This is accomplished in part by preprocessing such as the physical separation of blood presented here, but also requires biochemical approaches in the form of antifouling layers. Facilitating the binding of analyte to the exclusion of these non-specifics in a complex medium is non-trivial, yet we note there are innovative approaches such as polymer brushes^32,41–43^ an area actively being explored. Finally, further optimisation of the antibody functionalisation and the sensor nanofabrication techniques will continue to improve the sensitivity and reproducibility of our sensing platform.

We note the importance of our solution in the context of other work which describes the detection of biomarkers in blood. The key advantage of our system is that it is not a piecemeal approach to the problem of sensing in complex media but rather a ‘start-to-finish’ sensing platform which is completely passive. This, paired with the capacity for multiplexing within a single channel and the stability of the system, enables this sensing platform to form the basis of other medical assays.

## Methods

### 3D printing

Computer-aided design models were generated for printed components using the Autodesk Inventor (Autodesk, USA) software. The designs were transferred to a Formlabs Form3+ resin printer (Formlabs, USA) and printed in Tough 2000, a resin composed of urethane dimethacrylate and methacrylate monomers^32,43,44^ (Formlabs, USA). The components were then submerged in isopropanol, agitated until excess resin was removed then dried under nitrogen. The parts were cured at 70 °C under 450 nm lamps.

### Cartridge

The cartridge consists of three main components printed via 3D stereolithography printing. The top component contains an inlet into which 0.5 mL of blood solution is added. There is an indentation which fixes a 16 mm nitrile O-ring and a commercially available plasma separation membrane (Vivid^TM^, USA) disc, an asymmetric polysulfone which captures the cellular components of blood in larger pores on the exposed side whilst permitting the flow of blood plasma and smaller proteins through to the device^32,33,44^.

As these channels exit the recess bordered by the O-ring, they are sealed by tape typically used for the sealing of biochemical assays (*Thermo Scientific*). The sensing chip is held in place by a recess in the bottom component. A seal is achieved with the fluidic component using a double-sided tape from Microfluidic ChipShop. A rubber gasket cushions the sensing chip from the bottom component. The fluid covers the sensor then is pumped via capillary action into a waste port fabricated into the microfluidic device. We include a schematic of all components of the sensing platform in the Supplementary Fig. 1.

### Micrographs

The samples were prepared by pipetting 50 µL of each solution onto glass microscope slides and letting dry completely. The smears were fixed in methanol then stained with Giemsa, a common haematology stain before being imaged using an optical microscope.

### Fabrication of chirped guided mode resonance sensors

The design and fabrication of chirped GMRs are described in previous work^31^. In brief, the gratings were fabricated by electron beam lithography on a 150 nm thick Si_3_N_4_ film on Borofloat Glass (Schott, Germany). The substrate was first cleaned using Piranha solution (a 3:1 mixture of 95% sulfuric acid and 30% hydrogen peroxide, both from Sigma-Aldrich). The samples were then sonicated in acetone and isopropanol before being dried under nitrogen gas. Resist (ARP-6200.13, AllResist GmbH) was spin-coated at 5000 rpm for 60 s and soft baked at 180 °C on a hotplate for 5 min. A charge dissipation layer was spin-coated at 2000 rpm followed by soft bake at 90 °C for 2 min. The pattern was exposed by an electron beam lithography tool (Voyager 50 KV, Raith GmbH) with a step size of 1 nm in x axis (perpendicular to the grating groove) and 4 nm in y axis (along the grating groove) at a dose of 145 μC/cm^2^ then developed in xylene for 2 min. Reactive ion etching was performed with a CHF_3_:O_2_ gas mixture at 58:2 sccm flow rate with an output power of 40 W; chamber pressure was kept at 1.9 e^−1^ mbar over 7 min. The residual resist was removed under sonication with 1165 solvent (MicroChem) for 12 min, followed by sonication in acetone and isopropanol for 5 and 3 min, respectively. The samples were finally dried under nitrogen stream.

### Functionalisation of chirped guided mode resonance sensors

The substrates were cleaned in Piranha solution for 5 min. A functionalisation protocol based on the polymerisation of dopamine under alkaline conditions was used^23^. The substrates were submerged in 2 mg/mL dopamine and pH 8.5 tris buffer solution for 30 min, creating films typically 2–3 nm thick^33,44,45^. The substrates were baked in a 150 °C oven for 30 min. These polydopamine films covalently bond biomolecules without the need for additional chemical crosslinker reagents or processing.

300 µg/mL solutions of Goat Antibody to Human CRP (Scripps Laboratories, GC019) and Goat IgG Isotype Control (Invitrogen 02-6202) were prepared in pH 7.4 PBS. Antibody solutions were spotted onto the substrate using a sciFLEXARRAYER S3 precision dispensing system (Scienion). After 30 min, the sensor was submerged in 1 mg/mL bovine serum albumin in PBS buffer to block unfunctionalized surfaces, minimising non-specific binding^33,45,46^. We chose bovine serum albumin as this was found to act as an effective blocking agent against non-specific binding of CRP on the reference sensors via quartz crystal microbalance with dissipation.

### Serum and blood samples

Serum samples were obtained from centrifuged inpatient blood samples at York Hospital. CRP concentration was quantified via immunoturbidimetry. Ethical approval was granted by Integrated Research Application System Project ID 315874, Health Research Authority 22/HRA/3039. Blood samples were obtained from healthy volunteers via fingerprick and venesection. Ethical approval was obtained from University of York Ethics Committee, KENT_DK202207.

## Supplementary information

Krauss_npjBiosensingSupplementary_Revision2

VideoofOperation

## Data Availability

The authors declare that the data supporting the findings of this study are available within the paper and its Supplementary Information files. Should any raw data files be needed in another format they are available from the corresponding author upon reasonable request.
