# Supplementary material for: A passive blood separation sensing platform for point-of-care devices
Source: NPJ Biosens. 2025 May 2;2(1):19. doi: 10.1038/s44328-025-00038-x (PMC12048346; doi:10.1038/s44328-025-00038-x)
Supplement: Supplementary file 1 — Krauss_npjBiosensingSupplementary_Revision2 [file 44328_2025_38_MOESM1_ESM.pdf]

SUPPLEMENTARY INFORMATION

A passive blood separation sensing platform for point-of-care devices

Cameron Gilroy*[1,2], Callum D Silver[2], Casper Kunstmann-Olsen[2], Lisa M Miller[2], Steven K Johnson[2], Thomas F Krauss*[2]

[1] Hull York Medical School, Siwards Way, University of York, Heslington, York YO10 5DD

[2] School of Physics and Technology, University of York, Heslington, York, YO10 5DD

Cameron Gilroy*: cameron.gilroy@york.ac.uk

Thomas F Krauss*: thomas.krauss@york.ac.uk

1. CARTRIDGE DESIGN

The components which comprise the cartridge are shown in **Supplementary Figure 1 a)**. The 3D printed components were designed using Autodesk Inventor (Autodesk, USA) software then transferred to a Formlabs Form3+ resin printer and printed in Tough 2000, a resin composed of urethane dimethacrylate and methacrylate monomers (Formlabs, USA). The assembly is fixed with screws. A photograph of the assembled cartridge is shown in **Supplementary Figure 1 b)**.

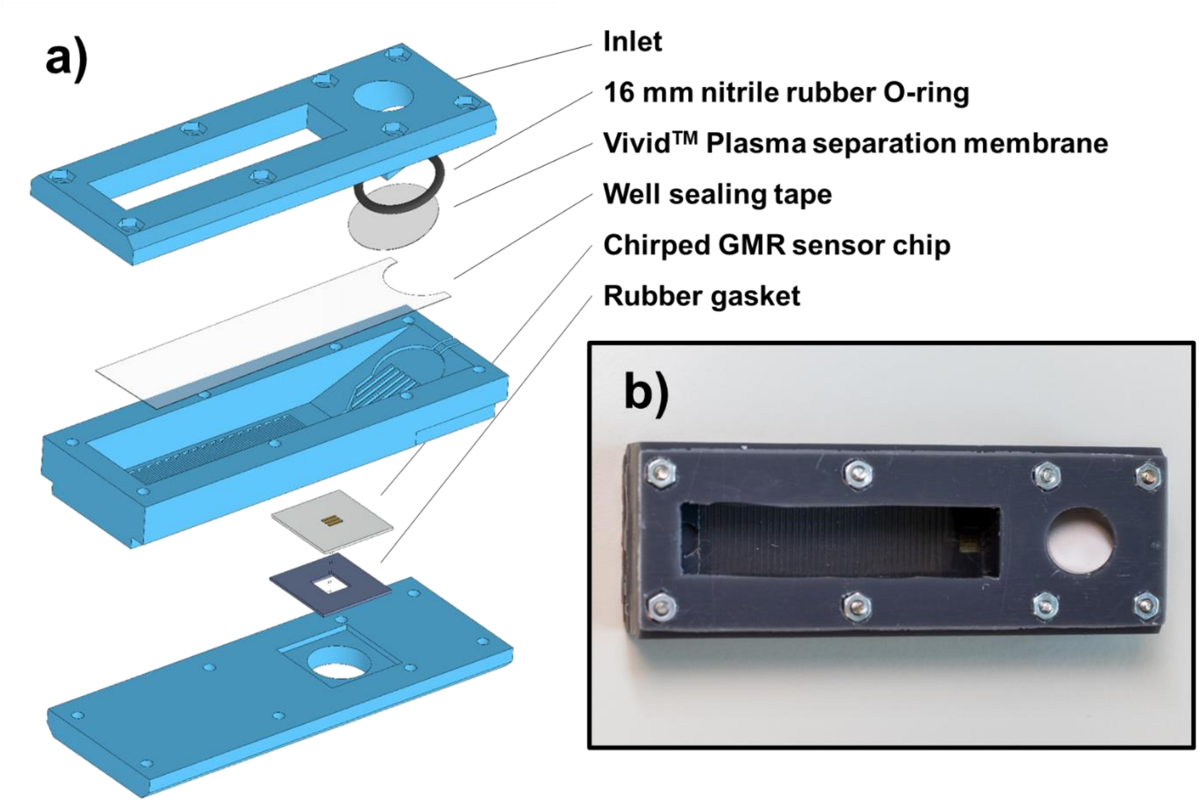

Supplementary Figure 1*:* The sensing cartridge. ***a)*** *Schematic diagram of the 3D printed device and components.* ***b)*** *Photograph of the assembled sensing platform.*

Printing time was approximately 1 hour, using 31.72 mL of resin. We estimate the cost of printing each cartridge to be £4.50 assuming £144 per litre of printing resin. Mass production techniques would clearly reduce time and cost.

2. DETECTION BOX

We use a custom-made 3D printed box which contains all required optical components as our detection unit. A schematic and photograph of this box is shown in **Supplementary Figure 2.** The image capturing and analysis protocol has been described in previous work[1].

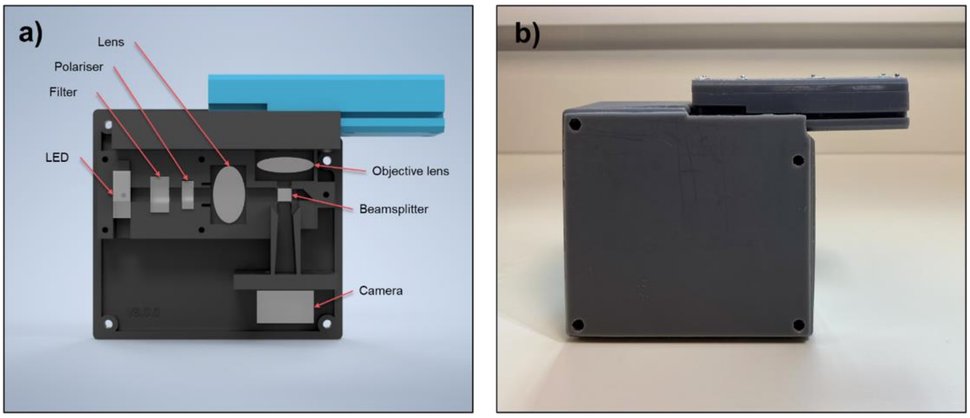

Supplementary Figure 2: Detection box. ***a)*** *Schematic of the custom-made detection box with optical components.* ***b)*** *photograph of the box with cartridge inserted.*

3. FLUORESCENCE

To illustrate the viability of the sensing method, we show the differential binding of the target molecule to the antibody and isotype functionalised sensors. To this end, we label CRP with a fluorophore, *Alexa Fluor 647 Dye (Thermofisher)*: to a tube containing 360 uL sodium bicarbonate buffer (0.1 M, pH 8.3) was added CRP (100 uL, 2 mg/mL, 17 uM) followed by Alexa647-NHS (40 uL, 160 uM). The resultant solution was shaken at room temperature for 1 hour. The excess reagents were then separated from the labelled protein using an Amicon® Ultra Centrifugal Filter, 10 kDa MWCO, washing twice with PBS. The labelled protein, CRP-Alexa647, was then recovered and stored as a PBS solution (17 uM). We show that this labelled CRP still binds with its antibody via quartz crystal microbalance.

We use a 2 mg/L solution of the labelled protein in PBS buffer, which is flowed over the antibody functionalised sensors. The surface was subsequently imaged via confocal fluorescence microscopy to assess the spatial distribution of the labelled CRP, as shown in **Supplementary Figure 3**.

The distribution of the CRP protein correlates well with the distribution of the spotted antibody and is uniform. The mean fluorescence intensity on the anti-CRP functionalised sensor is ~11 times that of the isotype reference channel and ~38 times that of the background borosilicate glass.

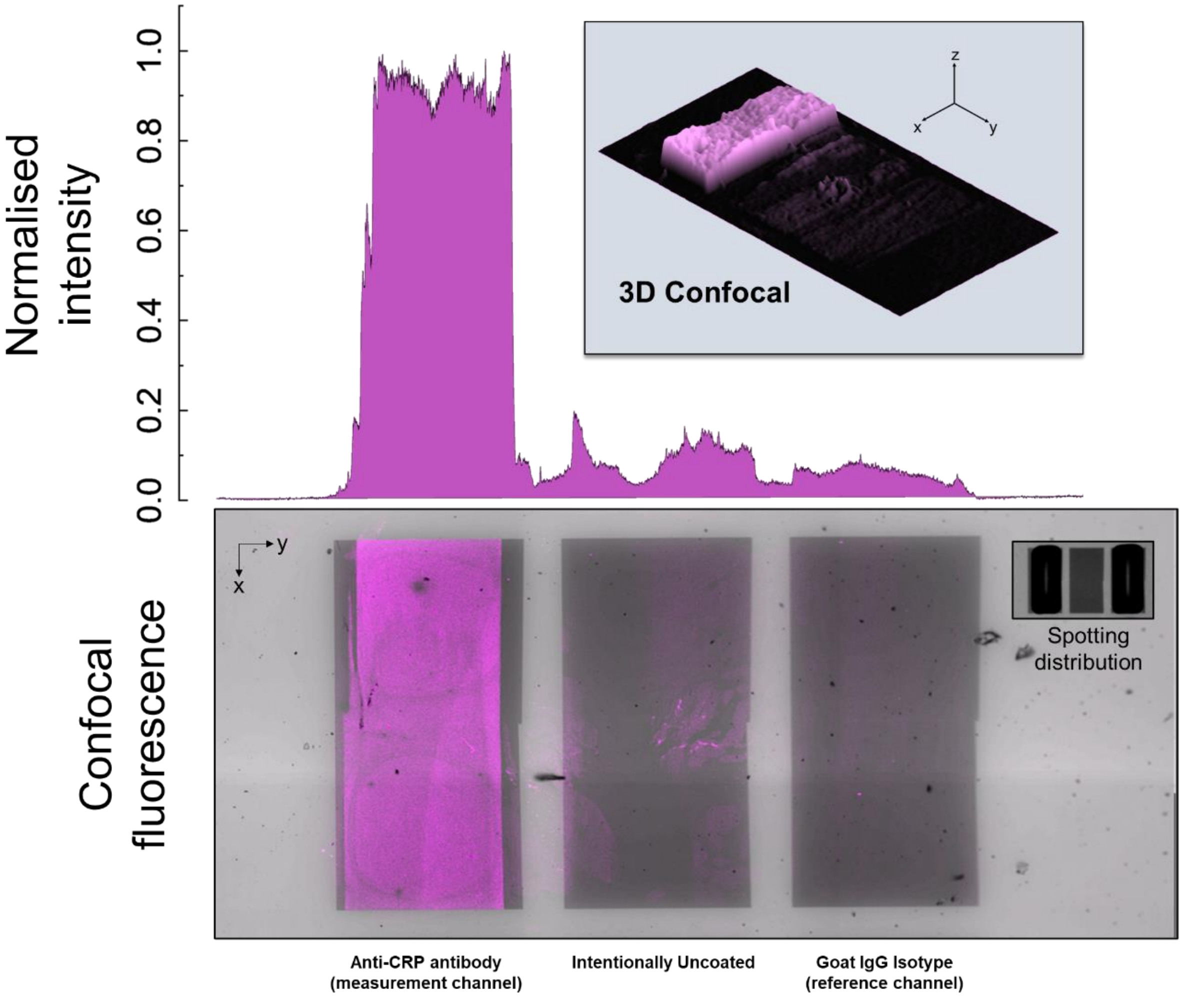

Supplementary Figure 3*: Confocal microscopy images of the GMR sensor functionalised with anti-CRP and goat IgG isotype antibodies: the bottom inset shows the spotting distribution. CRP labelled with an Alexa647 fluorophore was flowed over the sensor and fluorescence (in pink) was mapped onto the image. Top inset shows a topographical map of the fluorescence intensity. The integrated intensity across the x-axis was calculated and normalised.*

4. ISOTYPE CONTROL

Here, we follow the same protocol as is outlined in the main text, methods section. In brief, different channels of the sensing region are functionalised with antibodies. As an additional control, we coat both measured sensors with the isotype reference antibody which lacks specificity to the target antigen (C-reactive protein, CRP).

To the cartridge, we add a 1 in 30 solution of patient blood plasma which has been determined at the hospital site to have 113.3 mg/L CRP and measure the response, **Supplementary Figure 4**. The two isotype-functionalised sensors show very similar responses with their difference at 800 ~0.03 microns. In the main text, the shift for the same plasma solution gives a shift difference of ~7 pixels between measurement and reference channels.

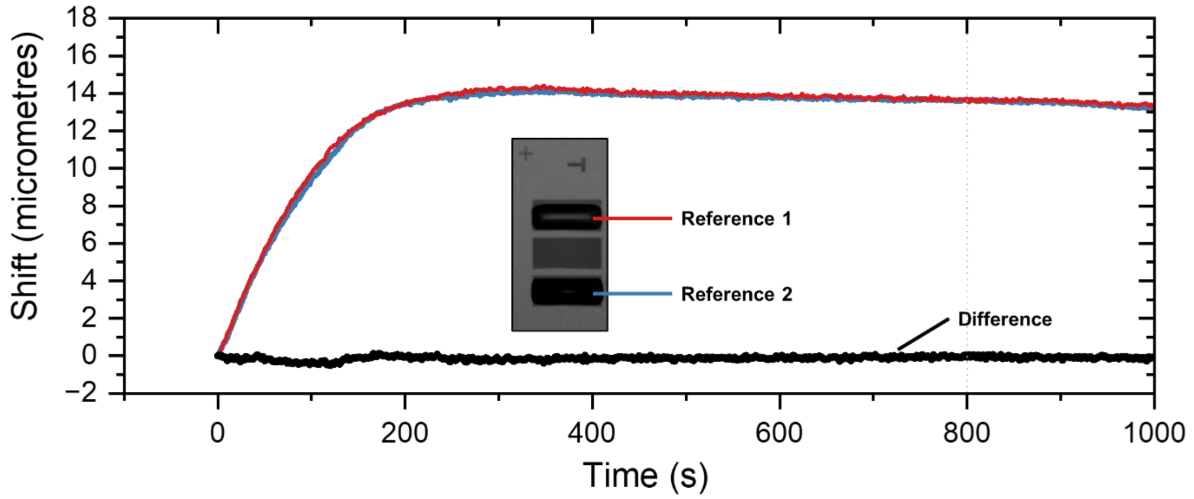

Supplementary Figure 4: *Response when both sensors are functionalised with the isotype antibody. Their shift is very similar yielding a difference of approximately 0.*

5. REFERENCES

1 Kenaan, A. *et al.* Guided mode resonance sensor for the parallel detection of multiple protein biomarkers in human urine with high sensitivity. *Biosens Bioelectron* **153**, 112047 (2020). https://doi.org/10.1016/j.bios.2020.112047
